# A positive serum basophil histamine release assay is a marker for ciclosporin-responsiveness in patients with chronic spontaneous urticaria

**DOI:** 10.1186/2045-7022-2-19

**Published:** 2012-10-01

**Authors:** Kamran Iqbal, Kapil Bhargava, Per Stahl Skov, Sidsel Falkencrone, Clive EH Grattan

**Affiliations:** 1 St John’s Institute of Dermatology, St Thomas’ Hospital, London, UK; 2 Department of Dermatology, Odense University Hospital, Odense, Denmark

**Keywords:** Ciclosporin, Chronic urticaria, Basophil histamine release assay

## Abstract

The electronic records of 398 patients with chronic spontaneous urticaria (CSU) who had had a serum basophil histamine release assay (BHRA) performed as a marker of functional autoantibodies were audited. The BHRA was positive in 105 patients (26.4%). Fifty eight were treated with ciclosporin because they were H1 anti-histamine unresponsive. CSU patients with a positive BHRA were more likely to respond clinically (P<0.001) and to have raised thyroid autoantibodies (P<0.02) than those with a negative BHRA. The BHRA offers a useful predictive biomarker for a good response of H1 antihistamine-unresponsive CSU patients to ciclosporin.

## Findings

Functional factors including histamine-releasing autoantibodies have been identified by the BHRA in the sera of 20–30% of patients with CSU [1-3]. CSU patients with a positive BHRA also have a higher incidence of thyroid antibodies than those without [4]. A double-blind placebo controlled study of ciclosporin for chronic idiopathic urticaria showed that more patients with a positive BHRA responded to ciclosporin than those with a negative BHRA [5] but this observation is not widely known. If confirmed, the BHRA could be used as a biomarker to predict a good response to treatment with ciclosporin and provide additional justification for using this third-line immunosuppressive agent in CSU patients with antihistamine-unresponsive disease.

The electronic case records of CSU patients attending a specialist Urticaria Clinic at St John’s Institute of Dermatology, London, who had a BHRA performed on their sera by RefLab, Copenhagen (HR-Urtikaria Test®) [6] between November 2004 and March 2011 were reviewed retrospectively by one of the authors to identify those with H1 antihistamine-unresponsive CSU treated with ciclosporin and their response to it. None of the patients had an autologous serum skin test. The usual starting dose of ciclosporin was 4 mg/kg/d and the usual duration of treatment was between 3 and 4 months. Patients with other patterns of chronic urticaria including inducible urticarias (physical, cholinergic and other types), angio-oedema without weals and urticarial vasculitis were excluded. The global response to ciclosporin was designated as complete, partial or none based on the assessment made by their attending clinician. The time to respond to ciclosporin was also noted when this information could be gleaned from the notes. The time to onset of a complete or partial response was categorised as immediate (within days), early (within a month), late (from 1-3 months) and delayed (beyond 3 months). An increase in autoantibodies to thyroid peroxidase, thyroglobulin or both above the laboratory normal ranges was noted. Frequency associations were analysed by the *χ*^2^ test (with Yates’s correction applied for small numbers) and a probability of P<0.05 was considered significant.

For the BHRA, donor cells were obtained from blood bank buffy coat cells fulfilling the following criteria for optimal response: 1) anti-IgE induced histamine release > 30%; 2) a pool of 10 sera from healthy non atopic individuals releasing < 16.5% were included as negative controls and 3) pools of 10 positive sera being either low positive (20 – 25%), moderate positive (30 – 35%) or highly reactive (> 45%) were included as positive controls.

A diagnosis of CSU (with or without angio-oedema) was made in 398 patients. The BHRA was positive in 105 (26.4%). Fifty eight patients who were unresponsive to second generation H1 antihistamines at up to fourfold licensed doses, with or without montelukast, were treated with ciclosporin (14.6% of the total). The BHRA was positive prior to treatment in 27 and negative in 31 patients. Three of the BHRA-positive patients and four of the BHRA-negative patients were on prednisolone (dose range 5-30 mg daily) at the time of starting ciclosporin without satisfactory disease control. None of them had received prior treatment with immunosuppressive therapies, including methotrexate or mycophenolate mofetil.

Their response to ciclosporin is summarized in Table 1. Eighty one percent of the BHRA + patients responded completely to ciclosporin but only 19% of the BHRA- patients did so (P<0.001). There was a trend to an earlier response in BHRA + patients that was not significant (Table 2). A positive serum BHRA was associated with increased thyroid autoantibodies (P<0.02, Table 3).

**Table 1:** **Clinical response to ciclosporinin CSU patients withpositive and negative serumBHRA**

	**+ve BHRA**	**-ve BHRA**
Complete response	22 (81%)	6 (19%)
Partial response	3 (11%)	10 (32%)
No response	2 (8%)	15 (49%)

*χ*^2^ = 22.7, df = 2, P<0.001.

**Table 2:** **Time from starting ciclosporinto observing a clinicalresponse in CSU patientswith positive and negativeserum BHRA**

	**+ve BHRA**	**-ve BHRA**
Immediate (days)	5 (25%)	0 (0%)
Early (< 1 month)	12 (48%)	11 (69%)
Late (1-3 months)	8 (32%)	4 (25%)
Delayed (> 3 months)	0 (0%)	1 (6%)

*χ*^2^ = 5.7, df = 3, P<0.1.

**Table 3:** **Frequency association of thyroidantibodies in CSU patientswith positive and negativeserum BHRA**

	**+ve BHRA**	**-ve BHRA**
+ve thyroid antibodies	9 (37%)	1 (4%)
-ve thyroid antibodies	15 (63%)	21 (96%)

χ_Y_^2^ = 5.52, df = 1, P<0.02.

The results of this retrospective case record review confirm the earlier findings of a double-blind placebo controlled study of ciclosporin [5] in which 13 of 18 clinical responders but only 1 of 9 non-responders to ciclosporin had a positive BHRA (p<0.01) but in a larger number of CU patients. They also confirm the findings of Kikuchi et al. [4] who found that there was a positive association between antithyroid antibodies in the sera of CSU patients with whole serum induced histamine releasing activity on heterologous basophils. The data on timing of response to ciclosporin is not strong enough to reach statistical significance but is in keeping with the clinical impression that most patients with a positive BHRA not only respond completely but often very rapidly.

The practical importance of these observations is that the BHRA can be used to predict a complete response to ciclosporin in patients with antihistamine-unresponsive CSU and justify the risks of using it as a short-term third-line therapy. Furthermore the good response to ciclosporin seen in patients with a positive BHRA and the association with thyroid autoimmunity provide further support for the concept of autoimmune urticaria in a subset of patients with the ordinary presentation of CSU [7]. The partial response seen in 10/31 (32%) of patients with a negative BHRA is probably due to temporary stabilization of histamine release from mast cells and basophils during treatment only. By contrast, the complete disease clearance in 81% of the CSU patients with a positive BHRA as a marker of functional IgG autoantibodies would be in keeping with the reduction of serum histamine releasing activity seen within one month of starting ciclosporin [5] in addition to any direct inhibitory effect on mast cell histamine release.

There was insufficient detail in the electronic patient records to draw conclusions about the severity of CU with or without a positive BHRA or the degree of responsiveness of patients to H1 antihistamines before treatment with ciclosporin and this would be worth examining in any future prospective study. The proportion of CSU patients requiring ciclosporin is very similar to the proportion of patients who were antihistamine-resistant in another recent large series from Italy (13.6%) [8]. Forty seven percent of these responded well to a 10-day reducing course of prednisolone with subsequent satisfactory control of their disease with antihistamines alone and the remainder were treated with ciclosporin. It is unknown whether or not the oral steroids prescribed to the seven patients in our series influenced the subsequent response to ciclosporin. Even if accurate information had been available, the numbers of BHRA positive and negative patients would have been too small to draw any meaningful conclusions.

The main limitations of this study are the retrospective data collection and the extraction of relevant information relating to the time to clinical response from the clinic letters. The researcher involved in collecting the clinical data from the patients’ electronic patient records (KI) was not involved with the care of any of the patients included in the analysis and therefore had no bias towards a particular outcome. The proportion of CU patients with a positive BHRA on the HR-Urtikaria® test is similar to a much larger series from patients with CSU sent to RefLab for testing by clinicians in different countries across Europe [6].

## Competing interests

Dr P Stahl Skov is an advisor to RefLab, Copenhagen.

## Authors’ contributions

KI examined the clinical records. KB co-ordinated the clinical audit. PS supervised the laboratory work. SF provided assistance with the BHRA. CEHG supervised the audit and wrote the manuscript. All authors have read the manuscript and contributed to the final revision.
